# Growth parameters and food frequently consumed by Basotho children aged 6–24 months old at Maseru and Leribe districts of Lesotho: A cross‐sectional study

**DOI:** 10.1002/pdi3.2503

**Published:** 2024-08-13

**Authors:** Mothusi Nyofane, Malebusa Rapotsane, Mohlotsane Moshao

**Affiliations:** ^1^ Department of Nutrition National University of Lesotho Maseru Lesotho

**Keywords:** complementary feeding, growth, stunting, undernutrition

## Abstract

Undernutrition in children remains a public health concern. Despite the global efforts to address undernutrition, Lesotho continues to bear the highest burden of childhood undernutrition. The study assessed the anthropometric measurements and dietary intake of children aged 6–24 months. A descriptive cross‐sectional study was conducted among 113 mother–child dyads attending clinic visits at Makoanyane Military Hospital (Maseru district); *n* = 50 and Motebang Hospital (Leribe district); *n* = 63. A structured sociodemographic and feeding practices questionnaires based on adapted World Health Organization (WHO) questionnaires were used. The usual food consumption was collected using an unquantified food frequency questionnaire. Anthropometric measurements and *z* scores computation were done as per WHO standard guidelines. Statistics included percentages for categorical variables and means for continuous variables. The percentage of continued breastfeeding was 54.0% in Maseru and 28.6% in Leribe districts. Complementary feeds were introduced at the mean age of 5.3 ± 1.0 (Maseru) and 5.2 ± 1.3 months (Leribe). In Leribe, 84.1% of children were consuming maize porridge every day while in Maseru, 68.0% of children were consuming commercial baby cereal every day. The prevalence of wasting was 10.0% (Maseru) and 20.6% with 14.3% of severe wasting (Leribe). A higher percentage of stunting was observed in Leribe (36.5%) than in Maseru (20.0%) (*p* < 0.001). The prevalence of moderate and severe stunting was 8.0% and 12.0% in Maseru and 20.6% and 15.9% in Leribe, respectively. The prevalence of stunting is alarmingly high in Leribe. The findings suggest an urgent need to strengthen maternal and child health and nutrition programs to ameliorate feeding practices and nutritional status.

## Graphical abstract

The prevalence of stunting was higher (36.5%) among children in the Leribe district than children in Maseru (20.0%); *p* < 0.001. A low rate (28.6%) of continued breastfeeding was observed in Leribe. In Leribe, 84.1% of children were consuming maize porridge while in Maseru, 68.0% of children were consuming commercial baby cereals.

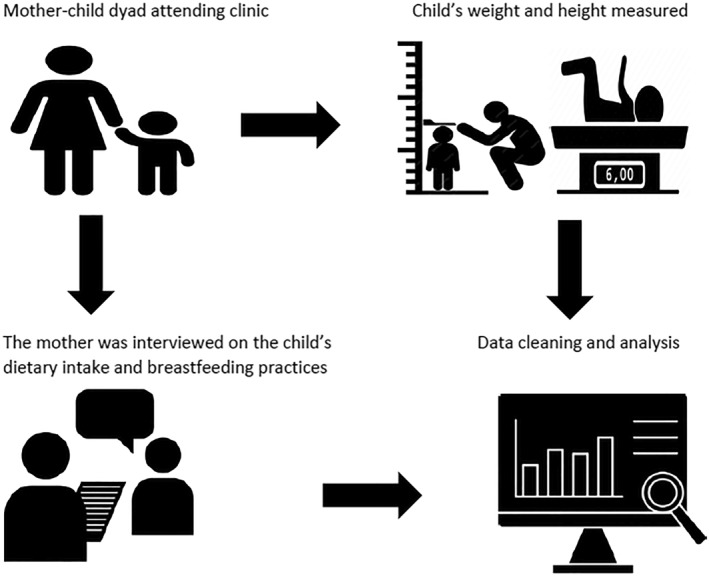

## 1 INTRODUCTION

Undernutrition is one of the significant public health challenges among children aged 6–59 months in low‐income countries, including Lesotho. Undernutrition poses a significant threat to children's overall health and well‐being by predisposing children to a greater risk of morbidity and mortality.^1^ Approximately 45% of all mortalities among children aged 6–59 months are attributed to undernutrition.^2^ Undernutrition is a predominant problem in various infections such as HIV. The interaction between undernutrition and infections can create a potentially lethal vicious cycle of catalyzing illness progression and deteriorating nutritional status.^3^

In 2020, it was estimated that 149.2 million children under five years suffered from stunting, while 45.4 million suffered from wasting.^1^ Unfortunately, Sub‐Saharan African countries, including Lesotho, bear the highest childhood undernutrition burdens. In the case of Lesotho, the prevalence of undernourishment has increased significantly from 13.7% to 34.7% from 2004/2006 to 2019/2021.^4^ Recent surveys indicated that Lesotho faces the double burden of malnutrition, with percentages of wasting and overweight computed to be 2.1% and 6.6%, respectively,^5^ and stunting estimated at 33.2%.^6^

Suboptimal nutrition in the first 1000 days of life is detrimental to the cognitive and physical health of the child, which could lead to reduced educational performance and economic productivity in adulthood.^7^ Numerous efforts have been made at the global and national levels to address malnutrition; however, we are still far from a world without malnutrition.^1^ Lesotho, in particular, has made significant strides toward progress in the fight against child undernutrition. Despite these global efforts, stunting, wasting, and being underweight remain vital challenges to the global and national health systems.

The nutritional status of children can be directly or indirectly associated with various factors, including low family income, low maternal education, breastfeeding, and poor dietary diversity.^8^ Other studies have associated food intake shortages with decreased production, poor food distribution channels, poverty, and illness with childhood malnutrition in developing countries.^9^ According to the Ministry of Health (Lesotho),^6^ the feeding practices of only 11.0% of children aged between 6 and 24 months meet the minimum standards of infant and young child feeding practices. Given this, the current study assessed the anthropometric measurements and dietary intake of Basotho children aged 6–24 months at the two selected health facilities in Lesotho.

## 2 METHODS

### 2.1 Study setting and participants

The study was conducted in Makoanyane Military Hospital (MMH), located in the district of Maseru, northwest of Lesotho. Maseru is the capital city with the largest urban area of Lesotho and boundless economic opportunities for all age groups, however, the under five child mortality rate is high at 76.3 per 1000 live births. Motebang Hospital is the district health facility in Leribe district, northwest of Lesotho. Leribe is dominated by many rural areas with livelihoods depending on agriculture and a higher under five mortality rate of 79.1 per 1000 live births. The mother–child dyad who went to their clinic visit during the day that the study was conducted were invited and recruited to the study. The interviewer ensured that the daily activities at the facilities were not interrupted.

### 2.2 Study design and population size

**FIGURE 1:**
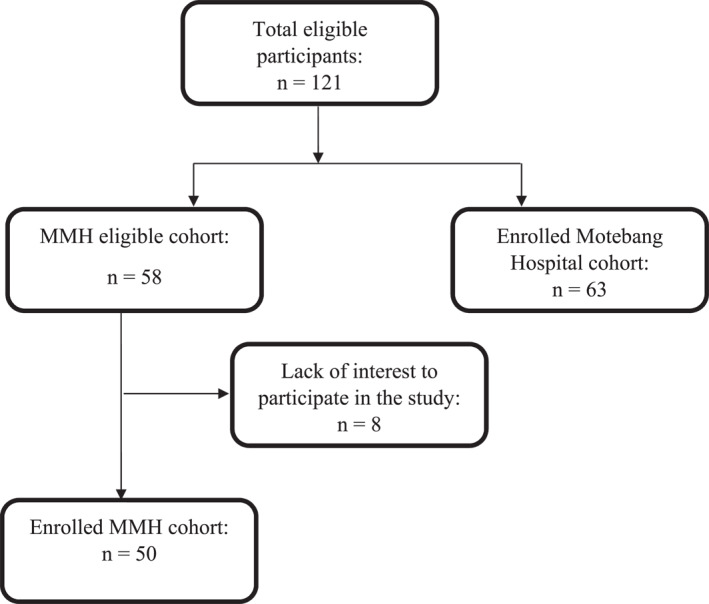
Study cohort. MMH, Makoanyane Military Hospital.

The study employed a descriptive cross‐sectional domain and convenience sampling to explore the nutritional status and feeding practices of children aged 6–24 months. Exclusion criteria included the inability to obtain written informed consent for any reason, caregivers under the age of 18 years and children with developmental abnormalities or severe medical conditions. The outcome variables were anthropometric measurements and modifiers included dietary intake and breastfeeding practices. The study anticipated to study overall 120 mother–child dyads in both health facilities (*n* = 60 per facility). At MMH, of the 58 participants who presented at the clinic, only 50 participants were recruited and interviewed (response rate of 86.2%). Eight participants did not offer consent to participate and, therefore, were not interviewed. About 63 mother‐child dyads were recruited and interviewed at Motebang Hospital. Overall, a total of 113 mother–child dyads were investigated in the present study (Figure 1).

### 2.3 Data collection methods

Participants were invited, and introduced to the study and thereafter, written informed consent was obtained. The data were collected in May 2022. One‐on‐one interviews were conducted to gather data on sociodemographic variables and feeding practices. Questionnaires were administered in the local language (Sesotho). The study used a self‐designed structured sociodemographic questionnaire and the structured and pre‐tested feeding practices questionnaire based on adapted World Health Organization (WHO) questionnaires.^10^ The descriptive dietary data on the usual food consumption of children was gathered using the unquantified food frequency questionnaire (FFQ), which covered food consumed in the past 7 days. FFQ entailed four answer options: every day, most days, once a week, and never. It has been used previously in other studies.^11^ The self‐designed data sheet was used to code anthropometric measurements. Measurements done were the child's weight, length and mid‐upper arm circumference (MUAC). The WHO standard procedures were followed when doing anthropometric measurements.^12^ Measurements were taken twice, and the mean value was used to ensure intra‐observer reliability. The third measurement was done if the two values differed by 0.05 cm or 0.05 kg. The data was managed and prepared for analysis using Microsoft Excel.

### 2.4 Data processing and statistical analysis

The outliers and *z* scores outside the range of the reference population of less than −3 and above +3 were checked and corrected in the event of a mistake in data capturing. The INTERGROWTH‐21^st^ (for birth anthropometry measurements) and WHO Anthro Survey Analyzer were used to compute *z* scores: weight‐for‐length z score (WLZ), weight‐for‐age z score (WAZ), length‐for‐age z score (LAZ), and MUAC‐for‐age z score (MUACZ). The study used WHO guidelines to define wasting, underweight, stunting and acute malnutrition as WLZ, WAZ, LAZ, and MUACZ less than −2 standard deviations (SD). The *z* scores less than −3 SD were defined as severe wasting, underweight, and stunting. The descriptive analyses were performed using SPSS version 25. Descriptive statistics included frequencies and percentages for categorical variables and means and standard deviations for continuous variables. Due to the demographic differences between studied districts, further analysis was done, and that was comparison between Maseru versus Leribe, using the Chi‐squared and Fisher exact tests for categorical data and independent *t*‐test for continuous data. Tests were performed at a 5% level of significance.

## 3 RESULT

A total of 113 mother–child dyads were investigated in the study. The mean age of study children was 14.2 ± 8.7 months in Maseru and 24.0 ± 5.8 in Leribe (Table 1). Overall maternal mean age was 28.4 ± 5.7 years, with 76.1% of the mothers married. The majority (71.4%) of mothers in Leribe had attained any secondary schooling, and in Maseru, 52.0% had attained post‐school education. Further comparison analysis showed a significant difference between the two districts (*p* < 0.001); any secondary schooling: 40.0% versus 71.4%, and post‐school education: 52.0% versus 6.4% for Maseru and Leribe, respectively. Overall, 69.9% of mothers were unemployed, of which a high percentage (84.1%) was of Leribe. Further, most (88.9%) mothers from Leribe district had low monthly household income ranging from M 0–M 2000.00 (Maloti). The comparison analysis indicated lower household monthly income in mothers from Leribe than those from Maseru district (*p* = 0.001).

**TABLE 1:** Maternal and child characteristics.

Measures	Statistic	Study population	Maseru	Leribe	*p* value^a^
Sample size	*n*	113	50	63	
Age (months)	Mean ± SD	19.1 ± 12.3	14.2 ± 8.7	24.0 ± 5.8	**<0.001**
Sex					
Male	*n* (%)	57 (50.4)	23 (46.0)	34 (54.0)	0.400
Female		56 (49.6)	27 (54.0)	29 (46.0)	
Maternal age (years)	Mean ± SD	28.4 ± 5.7	27.8 ± 5.0	28.9 ± 6.4	0.128
Marital status					
Single	*n* (%)	27 (23.9)	8 (16.0)	19 (30.1)	0.080
Married		86 (76.1)	42 (84.0)	44 (69.9)	
Educational level					
Any primary schooling	*n* (%)	18 (15.9)	4 (8.0)	14 (22.2)	**<0.001**
Any secondary schooling		65 (57.5)	20 (40.0)	45 (71.4)	
Post‐school education		30 (26.5)	26 (52.0)	4 (6.4)	
Employment status					
Unemployed	*n* (%)	79 (69.9)	26 (52.0)	53 (84.1)	**<0.001**
Employed		34 (30.1)	24 (48.0)	10 (15.9)	
Monthly household income^b^	*n* (%)				**0.001**
M 0–M 2000		86 (76.1)	30 (60.0)	56 (88.9)	
M 2001–M 4000		3 (2.7)	1 (2.0)	2 (3.2)	
M 4001–M 6000		17 (15.0)	14 (28.0)	3 (4.7)	
M 6001–M 8000		6 (5.3)	5 (10.0)	1 (1.6)	
M 8000 and above		1 (0.9)	0 (0)	1 (1.6)	

*Note*: Significant difference is indicated by bolded *p* value.

Abbreviations: M, maloti; SD, standard deviation.

^a^ Further analysis was performed to compare the two facilities using Chi‐squared and Fisher exact tests. All tests were performed at a 5% level of significance.

^b^ Currency conversions: 1 Lesotho Loti = 0.053 US Dollar.

The feeding practices were based on maternal recall, and the findings are presented in Table 2. Overall, 95.6% of the study children were ever breastfed. The percentage of children who were on continued breastfeeding was 54.0% in Maseru and 28.6% in Leribe, and the comparison between the two districts showed a statistically significant difference (*p* = 0.006). For children no longer receiving breastmilk, breastfeeding was stopped from 7.0 ± 6.1 months in Maseru and 10.3 ± 4.2 months in Leribe. In this study population, the first complementary feeds introduced to children lacked protein‐rich foods, commercial baby cereal, fruits, and any milk and dairy products. In Leribe, 55.0% of children were first introduced to either maize meal or sorghum soft porridge and in Maseru, 40.0% and 46.0% of children were first introduced to soup and baby food in a jar or pureed, respectively. The findings on the first introduction of complementary feeds were statistically significant between the Maseru and Leribe districts (*p* < 0.001). Children were first introduced to complementary feeds at the mean age of 5.3 ± 1.0 months and 5.2 ± 1.3 months in Maseru and Leribe, respectively.

**TABLE 2:** Feeding practices of 6–24 month‐old children in Maseru and Leribe districts based on maternal recall.

Measures	Study population	Maseru	Leribe	*p*‐value^a^
Breastfeeding feeding practices, *n* (%)				
Ever breastfeed	108 (95.6)	46 (92.0)	62 (98.4)	0.999
Never breastfeed	5 (4.4)	4 (8.0)	1 (1.6)	
Currently breastfeeding	45 (39.8)	27 (54.0)	18 (28.6)	**0.006**
Breastfeeding cessation (months), mean ± SD	8.65 ± 5.2	7.0 ± 6.1	10.3 ± 4.2	0.115
Reasons for stopping breastfeeding, *n* (%)				**<0.001**
School or work	14 (12.4)	5 (10.0)	9 (14.3)	
Maternal HIV status	11 (9.7)	6 (12.0)	5 (7.9)	
Maternal ill‐health	6 (5.3)	2 (4.0)	4 (6.3)	
Maternal choice	12 (10.6)	9 (18.0)	3 (4.7)	
Family influence	11 (9.7)	0 (0)	11 (17.4)	
Perceived insufficient breastmilk	6 (5.5)	1 (2.0)	5 (7.9)	
The child did not want to eat other foods	8 (7.1)	0 (0)	8 (12.7)	
Age in months of the introduction of complementary feeds, mean ± SD	5.3 ± 1.2	5.3 ± 1.0	5.2 ± 1.3	**0.001**
First complementary foods introduced^b^, *n* (%)				**<0.001**
Protein‐rich foods	1 (0.9)	1 (2.0)	0 (0)	
Any soft porridge^c^	35 (31.0)	0 (0)	35 (55.0)	
Commercial baby cereal	1 (0.9)	0 (0)	1 (1.6)	
Potatoes	13 (11.5)	3 (6.0)	10 (15.9)	
Soup^d^	24 (21.2)	20 (40.0)	4 (6.3)	
Baby food in a jar/pureed	28 (24.8)	23 (46.0)	5 (7.9)	
Any vegetables	8 (7.1)	0 (0)	8 (12.7)	
Any fruits	1 (0.9)	0 (0)	1 (1.6)	
Any milk^e^ and dairy products	3 (2.7)	3 (6.0)	0 (0)	

*Note*: Significant difference is indicated by bolded *p* value.

Abbreviation: SD, standard deviation.

^a^ A further analysis was performed to compare the two facilities using the Chi‐squared and Fisher exact tests. All tests were performed at a 5% level of significance.

^b^ First food item is given to the child to complement breastmilk.

^c^ Maize meal porridge or sorghum porridge.

^d^ Either vegetable soup or soup with meat pieces.

^e^ Any milk included fresh animal, powdered, and sour milk.

Table 3 presents the findings on the usual consumption of food items covering seven consecutive days. Most (84.1%) of children from Leribe consumed stiff maize meal porridge (pap) daily and 54.0% of children from Maseru consumed sorghum porridge most days. Commercial baby cereal and oats were consumed daily by 68.0% and 44.0% of children in Maseru and never consumed by 71.4% and 81.0% of children from Leribe, respectively. A high percentage of children consumed formula milk (50.0%) and yoghurt (82.0%) daily in Maseru. Food enrichment with peanut butter and margarine was done in less than one‐third of children in both districts. In Maseru, 42.0% of children consumed any fruits every day and most days, respectively, and below a quarter of children consumed green vegetables. In Leribe, 55.6% and 34.9% of children were given green vegetables and any fruit every day.

**TABLE 3:** Usual consumption of food items by children aged 6–24 months determined by a 7‐day food frequency questionnaire.

Foods	Maseru, *n* (%)				Leribe, *n* (%)				
	Every day	Most days	Once a week	Never	Every day	Most days	Once a week	Never	*p*‐value^a^
Starches									
Soft maize meal porridge	0 (0)	3 (6.0)	1 (2.0)	46 (92.0)	9 (14.3)	7 (11.1)	6 (9.5)	41 (65.1)	**0.004**
Stiff maize meal porridge	22 (44.0)	11 (22.0)	3 (6.0)	14 (28.0)	53 (84.1)	5 (7.9)	1 (1.6)	4 (6.3)	**<0.001**
Soft sorghum porridge	1 (2.0)	27 (54.0)	11 (22.0)	11 (22.0)	31 (49.2)	9 (14.3)	3 (4.8)	20 (31.7)	**<0.001**
Commercial baby cereals	34 (68.0)	2 (4.0)	6 (12.0)	8 (16.0)	14 (22.2)	4 (6.3)	0 (0)	45 (71.4)	**<0.001**
Oats	22 (44.0)	13 (26.0)	9 (18.0)	6 (12.0)	2 (3.2)	6 (9.5)	4 (6.3)	51 (81.0)	**<0.001**
Bread	14 (28.0)	13 (26.0)	12 (24.0)	11 (22.0)	27 (42.9)	15 (23.8)	1 (1.6)	20 (31.7)	**0.002**
Rice	1 (2.0)	16 (32.0)	21 (42.0)	12 (24.0)	6 (9.5)	9 (14.3)	21 (33.3)	27 (42.9)	**0.019**
Noodle	12 (24.0)	25 (50.0)	6 (12.0)	7 (14.0)	5 (7.9)	8 (12.7)	3 (4.8)	47 (74.6)	**<0.001**
Animal‐based foods and meat products									
Eggs	18 (36.0)	23 (46.0)	7 (14.0)	2 (4.0)	8 (12.7)	34 (54.0)	6 (9.5)	15 (23.8)	**0.002**
Red meat	12 (24.0)	16 (32.0)	13 (26.0)	9 (18.0)	0 (0)	3 (4.8)	3 (4.8)	57 (90.5)	**<0.001**
Chicken	5 (10.0)	12 (24.0)	20 (40.0)	13 (26.0)	12 (19.0)	24 (38.1)	18 (28.6)	9 (14.3)	0.098
Liver	0 (0)	23 (46.0)	11 (22.0)	16 (32.0)	3 (4.8)	7 (11.1)	14 (22.2)	39 (61.9)	**<0.001**
Milk and dairy products									
Breastmilk	27 (54.0)	0 (0)	0 (0)	23 (46.0)	18 (28.6)	0 (0)	0 (0)	45 (71.4)	**0.002**
Formula milk	25 (50.0)	8 (16.0)	0 (0)	17 (34.0)	10 (15.9)	0 (0)	0 (0)	53 (84.1)	**<0.001**
Cow's milk	0 (0)	6 (12.0)	5 (10.0)	39 (78.0)	19 (30.2)	7 (11.1)	2 (3.2)	35 (55.6)	**<0.001**
Sour milk	1 (2.0)	0 (0)	5 (10.0)	44 (88.0)	4 (6.3)	5 (7.9)	0 (0)	54 (85.7)	**0.009**
Yoghurt	41 (82.0)	2 (4.0)	7 (14.0)	0 (0)	14 (22.2)	20 (31.7)	11 (17.5)	18 (28.6)	**<0.001**
Food items added to foods									
Peanut butter	4 (8.0)	9 (18.0)	8 (16.0)	29 (58.0)	6 (9.5)	5 (7.9)	0 (0)	52 (82.5)	**0.002**
Margarine	0 (0)	7 (14.0)	7 (14.0)	36 (72.0)	8 (12.7)	8 (12.7)	1 (1.6)	46 (73.0)	**0.006**
Fruits									
Any fruit^b^	21 (42.0)	2 1 (42.0)	2 (4.0)	6 (12.0)	22 (34.9)	18 (28.6)	11 (17.5)	12 (19.0)	0.069
Vegetables									
Green vegetables	7 (14.0)	6 (12.0)	0 (0)	37 (74.0)	35 (55.6)	17 (27.0)	5 (7.9)	6 (9.5)	**<0.001**
Miscellaneous									
Baby food in a jar/pureed	31 (62.0)	0 (0)	5 (10.0)	14 (28.0)	2 (3.2)	1 (1.6)	0 (0)	60 (95.2)	**<0.001**
Soup^c^	25 (50.0)	4 (8.0)	9 (18.0)	12 (24.0)	22 (34.9)	16 (25.4)	10 (15.9)	15 (23.8)	0.095
Tea	0 (0)	5 (10.0)	1 (2.0)	44 (88.0)	38 (60.3)	4 (6.3)	1 (1.6)	20 (31.7)	**<0.001**
Fizzy drinks	0 (0)	0 (0)	0 (0)	50 (100.0)	3 (4.8)	3 (4.8)	4 (6.3)	53 (84.1)	**0.033**
Fruit juice	19 (38.0)	15 (30.0)	4 (8.0)	12 (24.0)	21 (33.3)	11 (17.5)	0 (0)	31 (49.2)	**0.008**
Juice concentrate	38 (76.0)	9 (18.0)	0 (0)	3 (6.0)	0 (0)	0 (0)	0 (0)	63 (100.0)	**<0.001**

*Note*: Significant difference is indicated by bolded *p* value.

^a^ A further analysis was performed to compare the two facilities using the Chi‐squared and Fisher exact test. All tests were performed at a 5% level of significance.

^b^ Included orange, green, yellow or red fruit type.

^c^ Either vegetable soup or soup with meat pieces.

The growth parameters of children per district are presented in Table 4. At age between 6 and 24 months, the mean weights and lengths of children were 9.5 ± 2.2 kg and 11.3 ± 3.4 kg, and 75.4 ± 9.8 cm and 82.1 ± 12.1 cm in Maseru and Leribe, respectively. The *z* scores indices were −0.0 ± 1.6, −0.2 ± 2.2, and −0.2 ± 1.5 in Maseru and −0.1 ± 2.2, −0.8 ± 2.6, and −0.2 ± 2.1 in Leribe for WLZ, LAZ, and WAZ, respectively. The prevalence of moderate and severe stunting was 8.0% and 12.0% in Maseru and 20.6% and 15.9% in Leribe, respectively. The prevalence of stunting differed significantly between the districts, with higher percentages observed in Leribe (36.5%) than in Maseru (20.0%) (*p* < 0.001).

**TABLE 4:** Anthropometric measurements, *z* scores, and nutritional classification of study children aged 6–24 months in the Maseru and Leribe districts.

Measures	Maseru	Leribe	*p* value^a^
Anthropometric measurements and *z* score indices at birth, mean ± SD			
Birth weight (kg)	2978.1 ± 0.8	2910.5 ± 0.6	0.230
Birth length (cm)	48.1 ± 4.4	48.2 ± 3.8	0.101
Birth weight‐for‐length *z* score	−0.6 ± 0.3	−0.8 ± 2.1	0.278
Birth length‐for‐age *z* score	−0.5 ± 1.6	−0.7 ± 2.0	0.341
Birth weight‐for‐age *z* score	−0.7 ± 0.7	−0.9 ± 1.4	0.7851
Anthropometric measurements and *z* score indices at 6–24 months, mean ± SD			
Weight (kg)	9.5 ± 2.2	11.3 ± 3.4	**0.008**
Length (cm)	75.4 ± 9.8	82.1 ± 12.1	**0.042**
Mid‐upper arm circumference	14.2 ± 1.5	14.7 ± 1.3	0.520
Weight‐for‐length *z* score	−0.0 ± 1.6	−0.1 ± 2.2	**0.009**
Length/height‐for‐age *z* score	−0.2 ± 2.2	−0.8 ± 2.6	0.189
Weight‐for‐age *z* score	−0.2 ± 1.5	−0.2 ± 2.1	0.055
Mid‐upper arm circumference *z* score	−0.3 ± 1.2	−0.3 ± 1.0	0.555
Nutritional classification, *n* (%)			
Wasting	5 (10.0)	13 (20.6)	0.062
Moderate wasting	4 (8.0)	4 (6.3)	0.169
Severely wasted	1 (2.0)	9 (14.3)	
Stunting	10 (20.0)	23 (36.5)	**<0.001**
Moderate stunting	4 (8.0)	13 (20.6)	**<0.001**
Severely stunted	6 (12.0)	10 (15.9)	
Underweight	7 (14.0)	10 (15.8)	0.433
Moderate underweight	5 (10.0)	4 (6.3)	0.130
Severely underweight	2 (4.0)	6 (9.5)	
Moderate acute malnutrition	0 (0)	1 (1.6)	0.542
Overweight	3 (6.0)	5 (7.9)	0.169

*Note*: All tests were performed at a 5% level of significance. Significant difference is indicated by bolded *p* value.

Abbreviations: cm, centimeter; kg, kilogram; n/a, not applicable; NA, not available; SD, standard deviation.

^a^ Further analysis was performed to compare the two facilities using chi‐squared and Fisher exact test.

## 4 DISCUSSION

Our findings indicated that most mothers breastfed their children; similar findings have been reported in South Africa.^13^ Also, above a quarter of children in Leribe were on continued breastfeeding. The WHO guidelines for optimal feeding practices recommend that breastfeeding to be continued for up to 2 years or beyond.^14^, ^15^ Lesotho adopted this guideline many years ago; however, the percentage of continued breastfeeding was still low in the Leribe district. A better percentage of above 50% was reported in Maseru. Breastmilk contributes adequate nutrition, energy, proteins, essential fatty acids, and vitamin A to support the growth and development of the child through the first 1000 days of life.^15^ This study's reasons for breastfeeding cessation included the mother's return to school or work and family influence (only in the Leribe district). Similar reasons have been documented.^13^ Another reason for mothers who stopped breastfeeding was because of their HIV status; they feared that they would infect their babies. The WHO guideline updates on HIV and infant feeding recommend similar breastfeeding practices for HIV‐uninfected women and those living with HIV, with the provision of antiretroviral therapy.^14^

In both districts, children were introduced to complementary food early at 5 months; similar findings have been reported worldwide.^16^ The literature has shown that determinants of early introduction of solid foods include low maternal educational level and low socioeconomic status.^17^ The WHO 2023 guideline for complementary feeding of infants and young children recommends that solid, semi‐solid, soft foods, and liquids be introduced at 6 months (180 days).^15^ The soft maize meal or sorghum porridge was the most commonly introduced complementary feed in the Leribe district. A similar introduction of soft maize meal porridge has been reported to be common among South African mothers living in rural areas.^18^ Soft maize meal porridge is a bulky and low nutrient‐density food that is diluted with water to obtain the desired thin consistency.^18^, ^19^ Further, the high content of phytates in the maize meal may inhibit the absorption of iron and zinc.^19^ The findings indicated that protein‐rich foods, fruits and vegetables, milk and dairy products were not adequately introduced to this study's participants.

Findings on the usual consumption of foods showed that stiff maize meal porridge and soft sorghum porridge were mostly consumed every day or most days in both districts. Other studies have reported similar findings on maize meal and sorghum consumption.^5^, ^11^, ^20^ Maize meal and sorghum are staple foods for Basotho, so this may be the reason for everyday consumption. Bread was also the most consumed food, every day in Leribe and most days in Maseru. Researchers have argued that even though the fortification of commercial maize meal and flour was made mandatory in countries like South Africa and Lesotho, this has a small effect on child nutrition because children consume small quantities of these food items.^11^ A high percentage of children in Maseru consumed commercial baby cereals, yoghurt, formula milk, and baby food in a jar every day. Commercial baby cereals have been fortified with essential nutrients including iron and zinc needed for child growth and development. However, the use of these foods may be limited by their high cost. Milk and dairy products were poorly consumed in the Leribe districts with one‐third of children consuming cow's milk. Regarding animal‐source foods, consumption of eggs was common in both districts, liver was consumed on most days in Maseru, and above one‐third of children consumed chicken on most days in Leribe. The findings differed from previous studies that stated that little animal protein is included in children's diets.^5^, ^19^ Above 50.0% average of children in Leribe consumed green vegetables and above one‐third of children in Maseru consumed any fruit every day. The findings were in line with the previous report.^11^ The WHO recommends consuming a diversified diet; animal‐source foods, fruits, and vegetables for children aged 6–23 months and formula or animal milk feeds for children aged 6–23 months who are not breastfed for many reasons.^15^

The percentages of wasting and underweight reported in the Maseru and Leribe districts were higher than the 3.0% and 10.0% previously reported in the country, respectively.^6^ In Leribe district, the percentage of stunting was higher compared to the 31.0% as previously reported, whereas in Maseru district, the stunting rate was lower than the previously reported (30.0%). Furthermore, the percentages of severe stunting reported in the two districts were higher than the 11.0% reported in the country.^6^ Stunting indicates chronic malnutrition and it is irreversible after 2 years. Stunted children do not fully reach their potential physical growth and cognitive development and they are likely to have reduced learning and working capacity (productivity). Lesotho's 2025 target is to reduce stunting from 33.2% to 23.0%.^5^ The findings of the 2014 Lesotho demographic and health survey indicated an association between children's nutrition status and household wealth.^6^

The present study determined and successfully reported growth parameters and food intake of children in the first 1000 days, a critical period for increasing growth and development. The lack of data on exclusive breastfeeding was a drawback in this study. Also, the study was not longitudinal; therefore, growth outcomes over time were not possible to obtain. The small sample size was also a drawback to the study.

## 5 CONCLUSION

More effort is needed to support and promote continued breastfeeding, particularly in the Leribe district. A low percentage of children were consuming animal‐based foods, milk, fruits, and vegetables. The prevalence of stunting is alarmingly high in the Leribe district. The findings suggest an urgent need to strengthen maternal and child health and nutrition programs to ameliorate child feeding practices and nutritional status, particularly stunting. Future studies aiming to advance in a similar study should consider a high‐quality longitudinal investigation to develop a better understanding of childhood growth over time and the causes of stunting. Against the high prevalence of stunting, there is a need to investigate Basotho children's neurodevelopmental outcomes.

## AUTHOR CONTRIBUTIONS

**Mothusi Nyofane**: Conceptualization; methodology; validation; formal analysis; investigation; writing—original draft preparation. **Malebusa Rapotsane**: Conceptualization; methodology; investigation; data curation. **Mohlotsane Moshao**: Conceptualization; methodology; investigation; data curation.

## CONFLICT OF INTEREST STATEMENT

The authors declare no conflicts of interest.

## ETHICS STATEMENT

Approvals to conduct the study were obtained from the National University of Lesotho, Faculty of Health Science, Institutional Review Board and the Ministry of Health Lesotho, Research and Ethics Committee, with reference number ID172‐2022. Further approvals were obtained at the two selected health facilities. Caregivers were provided with relevant information, and they gave written informed consent for themselves and their children to participate in the study. There was no risk associated with participating in the present study. The study was conducted following the Helsinki Declaration.

## Data Availability

The data that support the findings of this study are available from the corresponding author upon reasonable request.

## References

[1] Malnutrition. United Nations Children's Funds (UNICEF). Accessed February 11, 2022. https://data.unicef.org/topic/nutrition/malnutrition/

[2] Malnutrition: Key Facts. World Health Organization (WHO). Accessed February 12, 2022. https://www.who.int/news‐room/fact‐sheets/detail/malnutrition

[3] Katona P , Katona‐Apte J . The interaction between nutrition and infection. Clin Infect Dis. 2008;46(10):1582‐1588. 18419494 10.1086/587658

[4] Food and Agricultrue Organisation (FAO) . The State of Food Security and Nutrition in the World 2022. 2nd ed. FAO; 2022.

[5] Lesotho Food and Nutrition Policy (LFNP), 2016‐2025. Government of Lesotho; 2016. Accessed August 12, 2024. https://faolex.fao.org/docs/pdf/les209966.pdf

[6] Lesotho Demographic and Health Survey 2014. Ministry of Health and ICF International; 2016. Accessed August 12, 2024. https://dhsprogram.com/pubs/pdf/FR309/FR309.pdf

[7] Vaivada T , Akseer N , Akseer S , Somaskandan A , Stefopulos M , Bhutta ZA . Stunting in childhood: an overview of global burden, trends, determinants, and drivers of decline. Am J Clin Nutr. 2020;112(suppl 2):777S‐791S. 32860401 10.1093/ajcn/nqaa159 PMC7487433

[8] Katoch OR . Determinants of malnutrition among children: a systematic review. Nutrition. 2022;96:111565. 35066367 10.1016/j.nut.2021.111565

[9] Gillani S , Shafiq MN , Bhatti MA , Ahmad TI . Impact of economic growth on child malnutrition in Pakistan: a time series analysis. IRASD J Eco. 2022;4(1):149‐163.

[10] Indicators for Assessing Infant and Young Child Feeding Practices: Part 2: Measurement. World Health Organisation (WHO). Accessed February 12, 2024. https://iris.who.int/bitstream/handle/10665/44306/?sequence=1

[11] Faber M , Laubscher R , Berti C . Poor dietary diversity and low nutrient density of the complementary diet for 6‐ to 24‐month‐old children in urban and rural KwaZulu‐Natal, South Africa. Matern Child Nutr. 2016;12(3):528‐545. 25138429 10.1111/mcn.12146 PMC6860079

[12] Physical Status: The Use of and Interpretation of Anthropometry, Report of a WHO Expert Committee. World Health Organisation (WHO). Accessed August 13, 2023. https://www.who.int/publications/i/item/9241208546 8594834

[13] Trafford Z , Jewett S , Swartz A , et al. Reported infant feeding practices and contextual influences on breastfeeding: qualitative interviews with women registered to MomConnect in three South African provinces. Int Breastfeed J. 2020;15(1):81. 32928259 10.1186/s13006-020-00315-7 PMC7489212

[14] World Health Organisation (WHO) . Consolidated Guidelines on the Use of Antiretroviral Drugs for Treating and Preventing HIV Infection: Recommendations for a Public Health Approach. 2nd ed. WHO; 2016. 27466667

[15] World Health Organisation (WHO) . Guideline for Complementary Feeding of Infants and Young Children 6–23 Months of Age. 1st ed. WHO; 2023. 37871145

[16] From the First Hour of Life: A New Report on Infant and Young Child Feeding. Accessed July 14, 2023. https://data.unicef.org/resources/first‐hour‐life‐new‐report‐breastfeeding‐practices/

[17] Wijndaele K , Lakshman R , Landsbaugh JR , Ong KK , Ogilvie D . Determinants of early weaning and use of unmodified cow’s milk in infants: a systematic review. J Am Diet Assoc. 2009;109(12):2017‐2028. 19942019 10.1016/j.jada.2009.09.003

[18] Du Plessis Lisanne M , Kruger HS , Sweet L . Complementary feeding: a critical window of opportunity from six months onwards. S Afr J Clin Nutr. 2013;26(3):S129‐S140.

[19] Faber M . Complementary foods consumed by 6‐12‐month‐old rural infants in South Africa are inadequate in micronutrients. Public Health Nutr. 2005;8(4):373‐381. 15975182 10.1079/phn2004685

[20] Mushaphi L , Mbhenyane X , Khoza L , Amey A . Infant‐feeding practices of mothers and the nutritional status of infants in the Vhembe District of Limpopo Province. S Afr N J Clin Nutr. 2008;21(2):36‐41.

